# Effects of User Characteristics on the Usability of a Home-Connected Medical Device (Smart Angel) for Ambulatory Monitoring: Usability Study

**DOI:** 10.2196/24846

**Published:** 2021-03-17

**Authors:** Noémie Chaniaud, Olga Megalakaki, Sophie Capo, Emilie Loup-Escande

**Affiliations:** 1 Centre de Recherche en Psychologie: Cognition, Psychisme et Organisations Université de Picardie Jules Verne Amiens France

**Keywords:** user characteristics, health literacy, home medical devices, usability study, remote health, home health

## Abstract

**Background:**

The Smart Angel home medical device allows ambulatory surgery patients to monitor their own health by taking their blood pressure and oxygen levels and answering a health questionnaire from home. Currently, this device is a prototype in the design phase, and no usability evaluation has been performed. This preventive device must be usable by patients with different profiles; however, it is important to select patients carefully to ensure their safety when using the device. As such, it would be interesting to know how to select or exclude patients. However, the links between user characteristics and the usability of this home medical device remain unclear.

**Objective:**

This study aims to better understand the links between certain characteristics of potential patients (ie, age, education, technophilia, and health literacy) and the usability (ie, effectiveness, efficiency, and satisfaction) of Smart Angel, as defined by the ISO 9241-11.

**Methods:**

We conducted an experimental study involving 36 participants investigating the effects of 4 patient characteristics (ie, age, education, technophilia, and health literacy) on usability, measured in terms of effectiveness, efficiency, and satisfaction. A mixed methods approach (subjective vs objective) using a variety of standard instruments was adopted (direct observation, video analysis, and questionnaires). First, to help participants project themselves into the real use of the Smart Angel device, they watched a scenario in a video. Second, the participants completed a set of questionnaires to show the extent of their health literacy level (Newest Vital Sign [NVS] and the Health Literacy Survey [HLS]) and then operated Smart Angel devices. Efficiency (ie, handling time) and effectiveness (ie, number of handling errors) measures were collected by video analysis. Satisfaction measures were collected by a questionnaire (System Usability Scale [SUS]). The qualitative observational data were coded using inductive analysis by 2 independent researchers specialized in cognitive psychology and cognitive ergonomics.

**Results:**

The results show a moderate and positive correlation between age and effectiveness (r=0.359; *P*=.03) and efficiency (r=0.357; *P*=.03). There is strong correlation between health literacy scored by the NVS and effectiveness (r=0.417; *P*=.01), efficiency (r=-0.38; *P*=.02), and satisfaction (r=0.45; *P*=.006). However, there is a weak correlation between technophilia and usability and no relationship between education level and usability.

**Conclusions:**

Our results show that literacy level and age are 2 important factors to consider when selecting future users of the Smart Angel device to ensure patient safety. This study also serves as an example promoting mixed methodologies in assessments of medical device usability that cannot be performed under real-world conditions.

## Introduction

### Background

Outpatient surgery has been on the rise in recent years. Performed operations are increasingly complex and dangerous for patients who have to manage their convalescence at home. The Smart Angel device is a home-connected medical device specifically designed to prevent postsurgical complications related to outpatient surgery. The purpose of this device is to facilitate the patient's return home by maintaining a link with the hospital. Upon returning home after an operation, the patient is required to use the device to send all their vitals 3 times a day for 1 week before returning the equipment to the hospital center. This postoperative follow-up may also enable patients to manage their convalescence better by avoiding all-too-frequent returns to emergency services or outpatient consultations [1].

Currently, this system is in an early design stage. Like any medical device, this tool must follow safety and quality standards [2] and usability standards [3] to meet the requirements of European Conformity (CE marking) for marketing. However, even today, the deployment of these connected medical devices is still hindered by their complexity of use, directly implying a lack of usability [4-6], thus impacting patient safety. With this in mind, Kortum and Peres commented, “A lack of usability may cost lives” [7].

### Usability

Usability is defined by the ISO 9241-11 [3] as “the degree to which a product can be used, by identified users, to achieve defined goals in an effective, efficient, and satisfactory manner, within a specified context of use.” This concept, which is still discussed by the scientific community, has 3 distinct dimensions: (1) *effectiveness:* the accuracy and completeness with which users achieve certain objectives; (2) *efficiency*: the relationship between accuracy and the resources used to attain it; and (3) *satisfaction*: user comfort and a positive evaluation of user interaction. Defined by these 3 dimensions, usability is linked to its context of use, characterized by 4 components: the task, the environment, the resources, and the users [3].

Despite the use of methodologies that involve the user in the design process [8-10], usability problems persist. There are 2 arguments in the literature that may explain this finding: (1) the lack of a standardized framework and method in usability studies [11-15], and (2) a lack of knowledge of the impact of the use context [16,17] on usability, in particular, user characteristics.

### User Characteristics

#### Age, Level of Education, Technophilia, and Health Literacy

Several researchers have recently investigated the relationship between user characteristics and the usability of connected devices in health care [11,18-22]. In particular, 4 user characteristics have been studied in the scientific literature: age [11,20,22-26], level of education [11,19,20], technophilia (ie, experience in information technology and previous experience with medical devices [11,23,27]), and health literacy [20,24,28,29]. In most studies, authors tend to agree on these interrelationships when investigating different devices. We detail these studies below.

#### Age

Many authors have examined the influence of age on the usability of connected devices in health care. Most of these authors concur on the influence of age on usability. For example, Georgsson and Staggers [11] investigated the usability of a diabetes management app running on a smartphone using the metrics of ISO 9241-11 (effectiveness, efficiency, and satisfaction). The authors found that the younger age group (30-49 years old) made fewer errors (ie, was more *effective*), was faster (ie, more *efficient*), and more *satisfied* (System Usability Survey [SUS] score of 88.33 vs 77.14) than the older group (50-69 years old). Sparkes et al [23] examined the usability of remote cardiac testing and found that the age of the participants impacted their ability to install the equipment. Younger subjects appeared to be more comfortable than older subjects. Jones and Caird [25] examined the use of a blood glucose meter and found that younger subjects had fewer difficulties and made fewer errors (ie, were more effective) than older subjects. Mykityshyn et al [26] also examined the use of a glucometer and found that young subjects were faster (ie, more efficient) than older subjects, regardless of the instruction format provided (written and drawn vs video). Van der Vaart et al [20] evaluated the usability of an application for monitoring the symptoms of 32 narcoleptics and found that usability (measured in terms of the number of tasks completed and problems encountered) was moderately and positively correlated with age and eHealth literacy level.

However, Liang et al [19] found no relationship between age and satisfaction as measured by the SUS score in their study on the evaluation of 7 health devices used by the general public (eg, connected watches), conducted with a sample of 388 participants. Similarly, Jensen et al [18] found no relationship between usability and the age of participants with respect to access and use of online health information. The authors explain that this result is probably due to the contrast in health literacy levels that would have taken precedence over the other variables.

#### Level of Education

The level of education is also a variable found in many usability assessments. However, to our knowledge, no studies have proven this link. Georgsson and Staggers [11], Liang et al [19], and Van der Vaart et al [20] have all found a lack of association between participants' level of education and usability (ie, effectiveness, efficiency, and satisfaction).

#### Technophilia

Differing results have been reported regarding the influence of technophilia—experience with information technologies (IT) and previous experience of medical devices—on usability. Georgsson and Staggers [11] found that those with more technology experience (what the authors call “IT/computer experience”) made fewer errors (ie, were more effective), were faster (ie, more efficient), and were more satisfied with the diabetes management application (+5 points for the SUS score). Conversely, Harte et al [27] conducted regression analyses between technology experience and SUS score and found no significant effect when evaluating a smartphone health app. Finally, Sparkes et al [23] showed that familiarity with the technologies seemed to have an influence on the correct installation of their device.

#### Health Literacy

##### Definition and Assessments

Health literacy is a user characteristic that can be expected to influence medical device usability [18,20,28,30]. Due to its multidimensionality, however, this characteristic is complex to define and difficult to assess. Sørensen et al [31] describe it as “an individual's knowledge, skills, motivation, and ability to identify, understand, evaluate, and use health information in decision-making in health care, disease prevention, and health promotion to maintain or improve lifelong quality of life.” However, this notion is often mentioned as a determinant to be considered in therapeutic education [32], prevention [33], therapeutic adherence, access to health information [18], and even recovery rate [32,34]. However, to our knowledge, no study has assessed the level of health literacy among the French population at the national level.

In terms of evaluation, health literacy is particularly difficult to measure for at least two reasons. The first reason concerns its multidimensional specificity [31]. The second reason is that health literacy is not related to socioeconomic criteria as might be intuitively assumed [35].

Currently, there are 2 main methods of measuring health literacy [36]: (1) questionnaire methods, by which an individual's abilities are assessed, and (2) self-reported methods, by which an individual's behaviors towards a health professional are directly observed. Currently, few tools exist in the French language compared to the 51 English-language instruments identified by Haun et al [37]. The most frequently used and cited instruments are part of questionnaire-based methods; they are the Test of Functional Health Literacy in Adults (TOFHLA) [38], the Rapid Estimate of Adult Literacy in Medicine (REALM) [39], the European Health Literacy Survey (HLS-EU) [31], and the Newest Vital Sign (NVS) [40]. However, these instruments have several limitations. Among these instruments, the REALM is more like a reading test than a comprehension test since participants are asked to read medical terms. The short version of the TOFHLA (ie, the Short Test of Functional Health Literacy in Adults [S-TOFHLA]), which assesses respondents' level of comprehension, seems more adapted to Swiss culture than to French culture [41] (indeed, direct reference is made to the Swiss health insurance system and the transmission of certain documents that do not apply to the French social security model). In addition, the validity of S-TOFHLA is currently the subject of some controversy due to inconsistencies in the interpretation of its component items [42]. Another instrument proposed in the literature, the NVS [40], shows a strong correlation (Cronbach α>.76) with the measurement of S-TOFHLA [43]. It also assesses some of the respondents' cognitive skills (reading, writing, comprehension, numeracy). Finally, the HLS-EU is based on the multidimensional literacy model of Sørensen et al [31]. This tool has identified important gaps in 8 European countries, as approximately 1 in 2 people reportedly have a problematic or inadequate level of health literacy [44].

##### Health Literacy and Usability

In the context of health technologies such as connected medical devices, which are increasingly becoming part of patient life, studies on the correlation between health literacy and usability are still rare or exploratory. Monkman and Kushniruk [21] propose an assessment of usability by considering health literacy through the design and validation of heuristic criteria. To do so, the authors adapted a set of existing guidelines for designing health-specific websites to make the content more understandable to users with a reliable level of health literacy. Using an electronic personal health record system, Czaja et al [28] were able to show that populations with low literacy levels had more difficulty using these tools. Kim and Xie [29] conducted a systematic review of articles examining the impact of low health literacy on the use of eHealth devices. Based on 74 studies, the authors conclude that the major barrier to accessing and using online health information for individuals with low literacy is strongly related to website usability. Jensen et al [18] found that participants with low levels of health literacy (as measured by REALM) used health technologies less. Those with low levels of health numeracy (as measured by TOFHLA) would have limited access to these technologies. This latter finding is consistent with those of Kaufman et al [24], who also concluded that low numeracy could be a barrier to using a telemedicine system. Chaniaud et al [30] showed that it is necessary to obtain a minimum level of prior health knowledge to use home medical devices. Finally, to our knowledge, no experimental studies have empirically characterized links between health literacy and usability in terms of efficiency, effectiveness, and satisfaction.

#### Study Objective

We have seen that the complexity of using medical devices resides essentially in usability problems [29], all the more so as they must be usable by patients with diverse profiles. In this sense, consideration of user characteristics, including age, education, technophilia, and health literacy, are important factors to consider in the design of a connected medical device such as Smart Angel for a patient's home. However, to our knowledge, no study involving all 4 of these characteristics has been conducted. Moreover, the relation between these characteristics and usability remains unexplored in the literature. Thus, the aim of this paper is to better understand the relationships between the 4 user characteristics of age, educational level, technophilia, and health literacy, and the usability (measured by effectiveness, efficiency, and satisfaction) of a connected medical device intended for a patient's home.

To do this, we formulated 4 hypotheses: (H1) older users will be less *effective, efficient*, and *satisfied* with the Smart Angel connected medical device than younger users [11,20,25,26]; (H2) users with a low level of technophilia (IT and medical device experience) will be less *effective, efficient*, and *satisfied* with the Smart Angel connected medical device than those with a high level of technophilia [11,23,27]; (H3) the level of education will not affect the *effectiveness, efficiency*, and *satisfaction* with the Smart Angel connected medical device [11,19,20]; and (H4) users with low levels of health literacy (as measured by NVS and HLS-EU scores) will be less *effective, efficient*, and *satisfied* with the Smart Angel connected medical device than those with high levels of health literacy [18,24].

## Methods

### Participants

We enrolled 36 participants for this study: 17 (47%) females and 19 (53%) males aged 20-64 (mean 40.75, SD 14.45) years. The inclusion criteria were that participants had to (1) have a 4G connection at home, (2) be under 70 years of age, (3) be eligible for outpatient surgery, and (4) not be at home alone. All participants were native French speakers and signed a consent form after being informed of the study's progress. The study was in line with the ethical recommendations of the Declaration of Helsinki. The participants were recruited on a voluntary basis, and no compensation was offered. Handover of the Smart Angel device took place at the participant's home or workplace.

### Materials and Measurements

The materials for this study included (1) the Smart Angel device, (2) personas and their scenarios, and (3) questionnaires (ie, 2 questionnaires assessing the level of health literacy, namely, the NVS and the HLS-EU; a questionnaire relating to sociodemographic data; and a questionnaire assessing satisfaction, namely, the SUS).

### The Smart Angel Device

**Figure 1:**
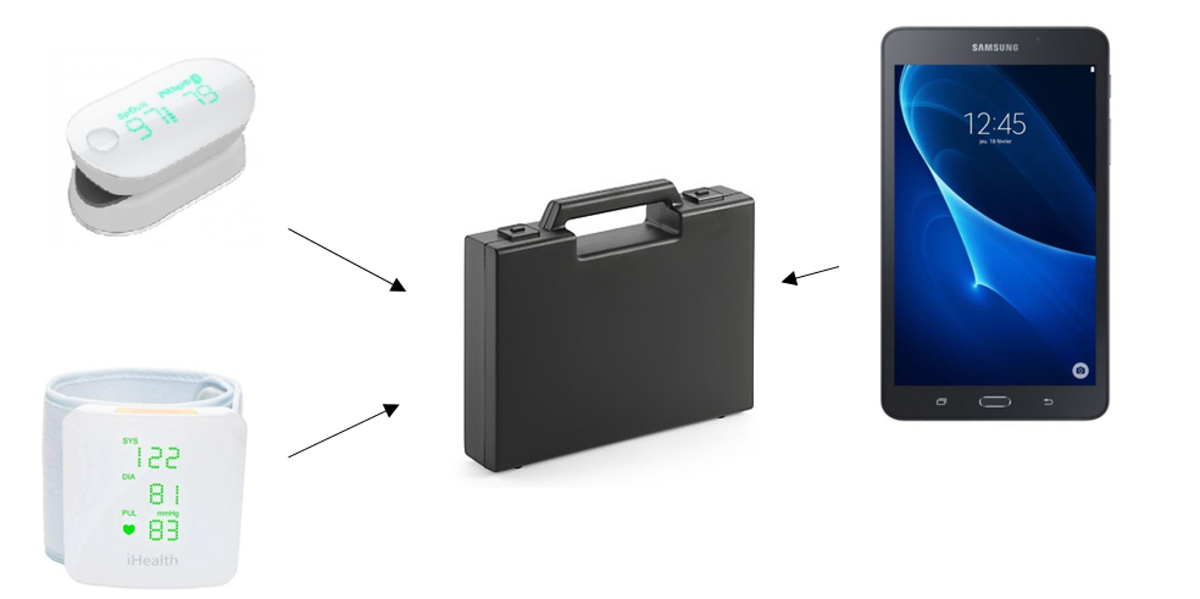
The Smart Angel components. Upper left: a pulse oximeter (iHealth Oximeter PO3); lower left: a wrist blood pressure monitor (iHealth BP7); right: a tablet with the Smart Angel application.

The Smart Angel device is designed by Evolucare Technologies. It consists of a Samsung 9-inch tablet with the Smart Angel application and 2 connected devices, a wrist blood pressure monitor (iHealth BP7) for blood pressure measurement and an oximeter (iHealth Oximeter PO3) for oxygen saturation and pulse measurement, which are available for the general public with European certification (Figure 1). To use the Smart Angel device, it is necessary to access the Smart Angel application and perform a digital medical “appointment” from a tablet application.

**Figure 2:**
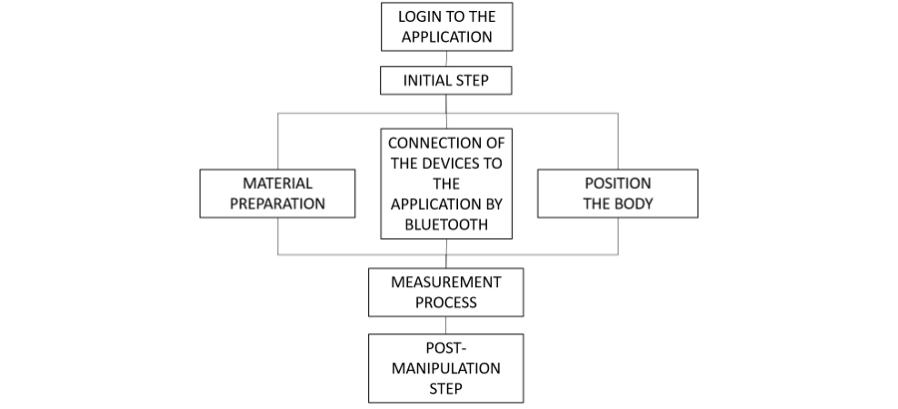
Schematic representation of the main steps in the use of the Smart Angel device.

The patient is given step-by-step instructions for connecting to and taking measurements with the blood pressure monitor and the pulse oximeter. The procedures for using the blood pressure monitor and pulse oximeter were built into the application; they include text and images for each step of the operation. For the 2 connected devices, the participant must first have a correct body position to then connect the equipment, install it correctly on themselves, start the measurement, and then remove and switch off the equipment. A schematic representation of this procedure is shown in Figure 2.

**Figure 3:**
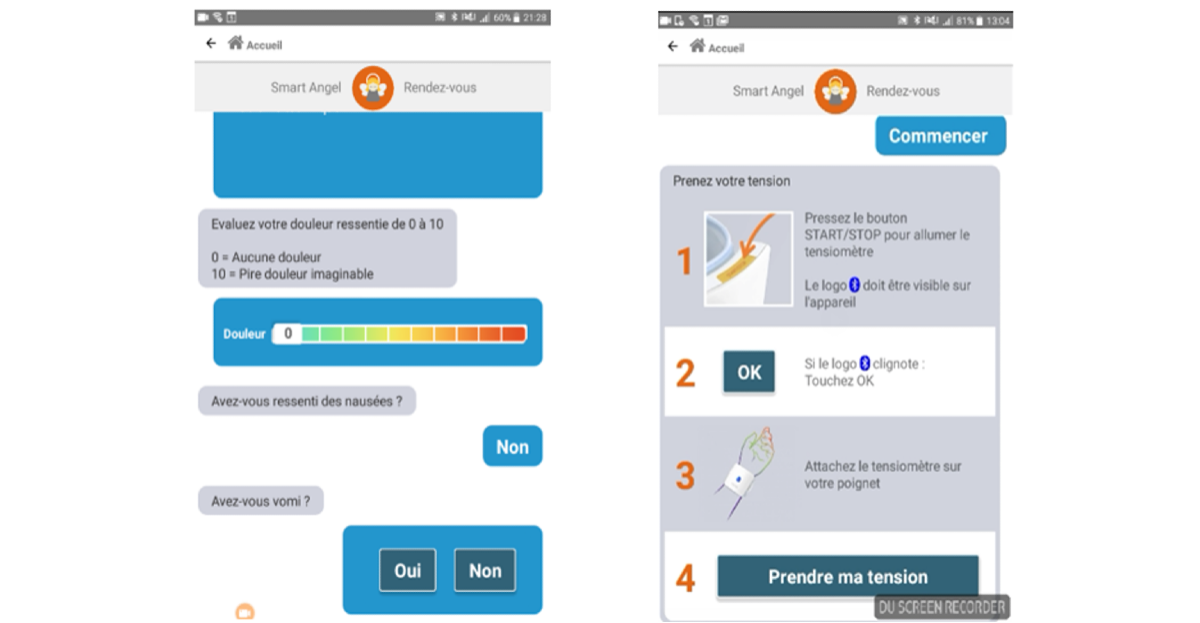
Screenshot of the Smart Angel application (Evolucare Technologies). Left: a form presenting an overview of the subjective state of health; right: the procedure for using the monitor.

Once the blood pressure or oxygenation measurement has been taken, the patient's health data are displayed on a colored gauge (from green to orange) according to the level of severity of the constant collected (Figure 3). Then, the user is presented with a questionnaire with various items related to general health, pain, sleep, and nausea. These items are presented either in simple-choice question format (eg, “How are you feeling today? Good, not good, not good at all”) or on a Likert scale (eg, “Rate your pain on a scale of 1 to 10”).

### Personas and Scenarios

We constructed 5 personas and their scenarios based on statistical surveys of outpatient surgical procedure types in France [45] and observations made in the field [46]. Generally used in the design phase, the personas method draws on the theory of mind and the theory of stereotypes and can provoke certain emotional states [47]. The personas scenarios were presented to the participants as audiovisual cartoons. All scenarios were constructed in the same way. Only the type of operation and the cause of the operation changed, according to each persona. An example of a persona is presented in Multimedia Appendix 1.

### Questionnaires

#### Measuring Health Literacy: Objective (NVS) and Subjective (HLS-EU) Assessments

Given the limited options of French-translated and validated health literacy questionnaires, we chose to use 2 health literacy questionnaires for a holistic view of this multidimensional skill: the NVS and the 16-item Health Literacy Survey (HLS-EU-Q16):

The French-translated [48] NVS [40] is a validated test assessing a patient’s ability to comprehend reading material and manipulate numbers (numeracy). Consequently, the NVS provides an objective assessment of health literacy level. Participants were asked to use an ice cream nutrition label to answer 6 questions (eg, “If I am allergic to peanuts, can I eat this ice cream?” Answer: “No, because the ice cream contains traces of peanut oil”). The total sum of the items (0-6 points) classified respondents into 3 categories: 0-1 point = inadequate health literacy; 2-3 points = problematic health literacy; 4-6 points = sufficient health literacy. The interitem reliability of the NVS in this study was good (Cronbach α=.883) [49].

The French-translated [50] HLS-EU-Q16 [31] is the short version of the HLS questionnaire. This version is composed of 16 items, 13 of which assess the 4 types of health literacy skills: the ability to access, understand, evaluate, and apply health information. Respondents were asked to rate their own ability to access information (eg, “Please rate, on a scale of very easy to very difficult, how easy is it for you to understand your doctor's or pharmacist's instructions on how to take your medication?”). Consequently, the HLS-EU-Q16 provides a subjective assessment of health literacy level. Answers are provided in 4 categories, on a 4-point Likert scale ranging from ”very easy“ to ”very difficult.“ To calculate the total score, the answers ”easy“ and ”very easy“ were assigned 1 point per item, and the answers *”*difficult“ and ”very difficult*“* were assigned 0 points per item. The total sum of the items (0-16 points) classified respondents into 3 categories: 0-8 points = inadequate health literacy; 9-12 points = problematic health literacy; 13-16 points = sufficient health literacy. The interitem reliability of the HSL-EU in this study was good (Cronbach α=.803) [49].

#### Sociodemographic Measurements (Age, Education Level, Technophilia, Etc)

This questionnaire includes the following personal details: age, gender, educational level, residential area, technophilia, and hospital experience. IT experience was measured by 2 items, adapted from Agarwal and Prasad [51], related to the participant’s use of and willingness to explore IT innovations (eg, “Which of these technologies do you use and how often?”*)*. On a 5-point Likert scale, the possible answers ranged from “never” to “very often.”

#### Measuring Usability (ISO 9241-11:2018)

##### Measuring Effectiveness

Effectiveness was measured by counting the number of manipulation errors, such as not putting the blood pressure cuff in the correct position. With respect to the use of the monitor, 5 categories of errors were identified: the participant (1) did not position the monitor correctly, (2) incorrectly directed the monitor toward the palm of the hand, (3) did not position the forearm correctly, (4) moved during the measurement, or (5) did not connect the monitor's Bluetooth to the tablet. Regarding the use of the pulse oximeter, 4 categories of error were identified: the participant (1) did not position the oximeter the right way, (2) did not insert the finger as far as the sensor, (3) removed the oximeter too early during the measurement, or (4) did not connect the Bluetooth from the oximeter to the tablet. With the tablet, 1 type of error was observed: the participant did not enter the appointment in the application. A scoring grid was used to identify these manipulation errors. When the participant made several attempts, we recorded the cumulative number of errors.

##### Measuring Efficiency

Measuring efficiency was based on the manipulation duration times of the various device tools for 3 measurements: blood pressure monitor manipulation, pulse oximeter manipulation, and total manipulation of the device, including the complete appointment. These times were measured from the time participants first touched the device (monitor, pulse oximeter, or tablet) to the time they turned it off after taking the measurement.

##### Measuring Satisfaction

Satisfaction was measured using the SUS. This ”quick and dirty“ questionnaire [52] consists of 10 items with 5 response options on a Likert scale (ranging from ”strongly disagree“ to ”strongly agree“), which allows for a subjective assessment of usability [53]. We used an adapted and validated version [54], in which we replaced the term ”system“ with the term ”medical device.“ Scores were calculated according to the recommendations of Brooke [52] and ranged from 0 to 100. Lower scores indicate low usability.

#### Procedure

The average duration of this experiment was 45 minutes. The selected participants did not come out of ambulatory surgery. Participants were first invited to choose among 5 proposed personas to allow them to project themselves into the needs of future users of the Smart Angel device [55]. The persona chosen had to be consistent with at least the participant’s age, profession, and previous surgery. Then, the researcher demonstrated the use of the Smart Angel device to the participant for about 3 minutes, sharing information about the correct manipulation of the device (eg, ”The monitor should always be at heart level“). Participants were asked to complete 3 questionnaires: the sociodemographic data questionnaire, the HLS-EU-Q16, and the NVS. Then they were asked to operate the Smart Angel device by taking a blood pressure measurement followed by an oxygen saturation measurement, and finally, by completing the general health questionnaire. There was no time limit for this. The participants were filmed during the process. The researcher could only intervene in the event of a technical problem (eg, battery problem). Finally, after the experiment, the participant had to respond to the SUS.

#### Data Analysis

The videos were analyzed using BORIS (Behavioral Observation Research Interactive Software) [56], which collected data on effectiveness and efficiency. Results were analyzed using SPSS software (version 22; IBM Corp). Each user characteristic was systematically compared to usability components, including effectiveness, efficiency, and satisfaction. For the health literacy measurement, we first analyzed the HLS-EU-Q16 result and then the NVS result. Bivariate correlations, ANOVAs, and Student *t* tests were performed when the sample met the homoscedasticity criteria, while nonparametric tests (Kruskal-Wallis and Mann-Whitney) were performed when the sample did not meet these criteria.

#### Interjudge Reliability: Objective Measures of Effectiveness and Efficiency

We used intraclass correlation (ICC) to verify interjudge reliability for quantitative data [57]. A 33% double coding of the collected video data was performed. The mean ICC measurement for total manipulation time (efficiency) was 0.978, with a 95% confidence interval of 0.918 to 0.994 (F_11,11_=45.436; *P*<.001). The mean ICC measurement (efficiency) for manipulating the monitor was 0.988, with a 95% confidence interval of 0.954 to 0.997 (F_11,11_=81.635; *P*<.001). The mean ICC measurement (efficiency) for manipulating the pulse oximeter was 0.956, with a 95% confidence interval of 0.838 to 0.988 (F_11,11_=22.955; *P*<.001). The mean ICC measurement (efficiency) for manipulating the tablet was 0.906, with a 95% confidence interval 0.652 to 0.975 (F_11,11_=10.688; *P*<.001). The mean measure of the number of manipulation errors (effectiveness) was 0.952, with a 95% confidence interval of 0.842 and 0.985 (F_11,11_=20.789; *P*<.001).

## Results

### Effects of User Characteristics on Usability

The correlations between user characteristics and usability components (ie, effectiveness = number of manipulation errors; efficiency = manipulation time; satisfaction = SUS score) were systematically analyzed (Table 1).

**Table 1:** Descriptive analyses of user characteristics, user experiences in health, medical devices, and technology (n=36).

Characteristics		Value	Average effectiveness, number of errors (SD)	Average efficiency, manipulation time in seconds (SD)	Average satisfaction, SUS score (SD)	
Age in years, mean (SD)		40.75 (14.45)	N/A^a^	N/A	N/A	
**Gender, n (%)**						
	Male	19 (52.8)	1.21 (1.27)	362.09 (144.16)	87.24 (11.18)	
	Female	17 (47.2)	2.06 (1.25)	373.91 (126.3)	81.03 (11.73)	
**Education, n (%)**						
	Secondary education	5 (13.9)	2.8 (1.64)	337.96 (89.67)	77 (9.75)	
	Higher education, 1st cycle	11 (30.6)	1.36 (1.1)	334.99 (106.25)	87.05 (13.82)	
	Higher education, 2nd cycle	11 (30.6)	1.64 (1.2)	412.95 (198.6)	82.73 (13.34)	
	Higher education, 3rd cycle	9 (25)	1.22 (1.3)	368.77 (80.88)	86.94 (5.97)	
**Residential area, n (%)**						
	Rural	6 (16.7)	1 (0.89)	362.92 (76.48)	88.75 (6.85)	
	Semi-urban	5 (13.9)	1.8 (2.05)	339.11 (72.3)	87 (11.37)	
	Urban	25 (64.9)	1.72 (1.24)	374.52 (154.78)	82.7 (12.62)	
**Persona chosen, n (%)**						
	Persona 1	8 (22.2)	*^b^	*	*	
	Persona 2	8 (22.2)	*	*	*	
	Persona 3	8 (22.2)	*	*	*	
	Personal 4	4 (11.1)	*	*	*	
	Persona 5	8 (22.2)	*	*	*	
**Health care experience with operations, n (%)**						
	Yes	32 (88.9)	1.59 (1.21)	376.18 (136.24)	85,39 (11.72)	
	No	4 (11.1)	1.75 (2.22)	299.55 (106.9)	75.62 (8)	
**Health care experience with outpatient operations, n (%)**						
	Yes	18 (50)	1.39 (1.33)	367.15 (142.09)	86.11 (11.8)	
	No	18 (50)	1.83 (1.29)	368.19 (130)	82,5 (11.66)	
**Health care experience with suffering from a chronic illness, n (%)**						
	Yes	11 (30.6)	1.27 (1.35)	386.68 (184.62)	81.14 (15.26)	
	No	25 (69.4)	1.76 (1.3)	359.3 (108.77)	85.7 (9.8)	
**Medical device experience with taking blood pressure, n (%)**						
	Yes	24 (66.7)	1.54 (1.32)	361.43 (131.26)	63.3 (11.22)	
	No	12 (33.3)	1.75 (1.36)	380.15 (145.06)	86.25 (12.9)	
**Medical device experience with blood oxygenation testing, n (%)**						
	Yes	5 (13.9)	0.4 (0.55)	309.16 (70.87)	89 (8.02)	
	No	31 (86.1)	1.81(1.3)	377.11 (140.31)	83.56 (12.12)	
**Information technology experience with ease of use of tablet/computer/telephone, n (%)**						
	Very comfortable	23 (63.9)	1.35 (1.23)	360.16 (120.21)	86.85 (11.24)	
	Relatively comfortable	11 (30.6)	2.27 (1.42)	401.34 (166.78)	78.18 (11.78)	
	Moderately comfortable	2 (5.6)	1 (0)	268.81 (33.95)	88.75 (5.3)	
	Rather uncomfortable	0 (0)	—^c^	—	—	
	Not at all comfortable	0 (0)	—	—	—	
**Frequency of use of technology, n (%)**						
	Very often (every day)	5 (13.9)	1 (1.22)	314.42 (48.65)	92.5 (6.85)	
	Often (several times a week)	12 (33.3)	1.25 (1.36)	364.46 (133.26)	90.42 (6.47)	
	Rarely (from time to time)	17 (47.2)	2 (1.27)	364.08 (99.16)	79.26 (11.38)	
	Very rarely (occasionally)	1 (2.8)	3 (—)	856.33 (—)	55 (—)	
	Never	1 (2.8)	1 (—)	244.8 (—)	85 (—)	

^a^N/A: not applicable.

^b^*Highly correlated to the ages of the participants.

^c^— Not available.

#### Age

**Figure 4:**
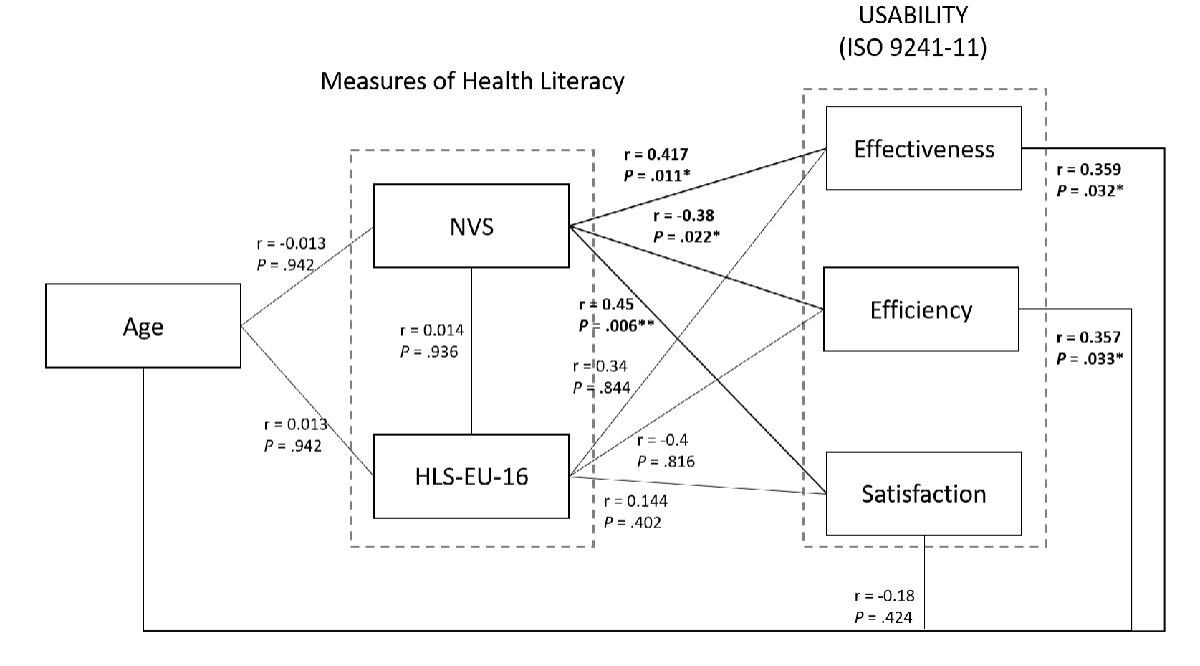
Schematic representation of correlations between age, measurements of participants' health literacy, and usability (ISO 9241-11). HLS-EU-Q16: 16-item European Health Literacy Scale; NVS: Newest Vital Sign. *P<0.5; **P<0.01.

The age of the participants (mean 40.75, SD 14.45, range 20-64 years) is significantly correlated (positively and weakly) with the number of manipulation errors (effectiveness: r=0.359; *P*=.03) and manipulation time (efficiency: r=0.357; *P*=.03). On the other hand, there was no significant correlation between age and SUS score (satisfaction: r=-0.138; *P*=.42). In addition, it is important to note that age is not correlated with the literacy level of the HLS-EU-Q16 (r=0.013; *P*=.94) or the NVS (r=-0.013; *P*=.94; Figure 4).

#### Technophilia

The IT experience of participants had no impact on the number of manipulation errors (effectiveness: F_5,30_=1.229; *P*=.32) or manipulation time (efficiency: F_5,30_=1.39; *P*=.26). On the other hand, there was a significant correlation between IT experience and SUS score (satisfaction: χ(3)=8.671; *P*=.03).

Moreover, previous experience of using medical devices that allow users to take their own blood pressure did not influence the number of manipulation errors (effectiveness: t_34_=0.443; *P*=.66), the manipulation time (efficiency: t_34_=0.39; *P*=.55), or the SUS score (satisfaction: Mann-Whitney U=104; *P*=.19). Previous experience of using medical devices for taking oxygen levels had a significant effect on the number of manipulation errors (effectiveness: t_34_=2.359; *P*=.02; η2=0.14), but this effect was not significant on the manipulation time (efficiency: t_34_=1.052; *P*=.30) or the SUS score (satisfaction: t_34_=-0.965; *P*=.34).

#### Educational Level

Educational level had no impact on usability in terms of the number of manipulation errors (effectiveness: F_3,32_=1.889; *P*=.15), manipulation time (efficiency: F_3,32_=0.698; *P*=.56), and SUS score (satisfaction: F_3,32_=1.076; *P*=.37).

#### Health Literacy

Systematic analyses were performed comparing the level of literacy (HLS-EU-Q16 and NVS) with each of the components of usability (effectiveness, efficiency, and satisfaction, as per the ISO 9241-11, 2018); Table 2 presents a descriptive representation of the results of the 2 health literacy questionnaires.

**Table 2:** Descriptive statistics of the 16-item European Health Literacy Survey (HLS-EU-Q16) and the New Vital Sign (NVS) questionnaires.

Statistic	HLS-EU-Q16	NVS
Mean (SD), range	12.97 (2.952), 5-16	4.17 (2.223), 0-6
Inadequate health literacy, n (%)	3 (8.3)	6 (16.7)
Problematic health literacy, n (%)	9 (25)	7 (19.4)
Sufficient health literacy, n (%)	24 (66.7)	23 (63.9)

##### HLS-EU-Q16 Questionnaire Results

There was no significant correlation between the results of the HLS-EU-Q16 and usability, either in terms of the number of manipulation errors (effectiveness: r=0.34; *P*=.84), manipulation time (efficiency: r=-0.40; *P*=.82), or the SUS score (satisfaction: r=0.144; *P*=.40). After correlation analysis, participants were clustered according to the HLS-EU-Q16 measures (Table 2), following the recommendations of Sørensen et al [31]. No intergroup differences could be observed between the HLS-EU-Q16 results and usability (Table 3) in terms of the number of manipulation errors (effectiveness: F_2.33_=0.277; *P*=.76), manipulation time (efficiency: F_2.33_=0.015; *P*=.99), and the SUS score (satisfaction: F_2.33_=0.483; *P*=.62).

**Table 3:** Analyses of the 16-item European Health Literacy Survey (HLS-EU-Q16) score according to usability (effectiveness, efficiency, and satisfaction; n=36).

HLS-EU-Q16 score classification group (n=36)	Effectiveness^a^, average number of errors (SD)	Efficiency^b^, average manipulation time (SD)	Satisfaction^c^, SUS^d^ score (SD)
Inadequate health literacy (n=3)	1.67 (2.08)	373.26 (88.76)	83.33 (3.82)
Problematic health literacy (n=9)	1.89 (1.27)	373.35 (98.53)	81.11 (14.53)
Sufficient health literacy (n=24)	1.50 (1.28)	364.84 (152.73)	85.62(11.3)

^a^ANOVA: F_2,33_=0.277; *P*=.76.

^b^ANOVA: F_2,33_=0.015; *P*=.99.

^c^ANOVA: F_2,33_=0.483; *P*=.62.

^d^SUS: System Usability Survey.

##### NVS Questionnaire Results

There was a significant mean-size correlation between the results of the French version of the NVS questionnaire and usability (Table 4) in terms of the number of manipulation errors (effectiveness: r=-0.417; *P*=.01), manipulation time (efficiency: r=-0.38; *P*=.02), and the SUS score (satisfaction: r=0.45; *P*=.006). In other words, the higher a participant's level of health literacy (measured using NVS), the fewer manipulation errors they made (ie, they are more effective), the faster they manipulate (ie, they are more efficient), and the higher their SUS score will be (ie, they will be more satisfied).

After analyzing the correlations, the participants were clustered according to the NVS measurements (Table 2), following recommendations [40]. No intergroup differences could be observed between NVS literacy and usability (Table 4) except for the number of errors (effectiveness: χ2=6.679; *P*=.04).

Further intergroup analysis (Figure 4) shows a significant effect between the inadequate-health-literacy and sufficient-health-literacy groups as a function of the number of manipulation errors (effectiveness: Mann-Whitney U=27; *P*=.02).

**Table 4:** Analyses of the New Vital Sign (NVS) results according to usability (effectiveness, efficiency, and satisfaction; n=36).

NVS score classification group (n=36)	Effectiveness^a^, average number of errors (SD)	Efficiency^b^, average manipulation time (SD)	Satisfaction^c^, SUS^d^ score (SD)
Inadequate health literacy (n=6)	2.67 (0.816)	463 (165.18)	77.08 (14.27)
Problematic health literacy (n=7)	1.71 (0.756)	387.72 (219.2)	80.71 (15.05)
Sufficient health literacy (n=23)	1.30 (1.43)	336.7 (75.79)	87.28 (9.07)

^a^Kruskal-Wallis test: χ^2^=6.679; *P*=.035, where *P*<.05 is significant.

^b^Kruskal-Wallis test: χ^2^=3.07; *P*=.21.

^c^ANOVA: F_2,33_=2.392, *P*=.11; Kruskal-Wallis test: χ^2^=2.618, *P*=.27.

^d^SUS: System Usability Survey.

## Discussion

### Principal Findings

This study’s objective was to better understand the relationships between 4 user characteristics (age, education, technophilia, and health literacy) and usability [3] (defined here as effectiveness, efficiency, and satisfaction) with regard to the use of the Smart Angel device. To do this, sociodemographic data were collected, literacy levels were investigated using the HLS-EU-Q16 [31] and the NVS [40], and usability measures were performed (errors and manipulation time, and SUS questionnaire).

We made 4 hypotheses that age (H1), technophilia (H2), and health literacy (H4) would have an impact on usability, while education level (H3) would not. Our first hypothesis (H1) was that older users would be less effective, efficient, and satisfied with the device compared to younger users. We can partially validate this hypothesis. The results show that the younger the individuals are, the less likely they are to make manipulation errors (ie, they are more effective) and the faster they manipulate the device (ie, they are more efficient). On the other hand, we did not observe any difference between the age of the subjects and the SUS score (satisfaction). All these results are in line with previous research [19,20,25,26]. Indeed, younger users are more effective (eg, Jones and Caird's glucometer [25]) and efficient (eg, Mykityshyn et al's glucometer [26] and Van der Vaart et al's application for narcoleptics [20]) compared to older users, with a positive and medium correlation [20]. However, younger users are as satisfied (SUS score) with the device as older users, which is consistent with the findings of Liang et al [19] while at variance with those of Georgsson and Staggers [11].

Our second hypothesis (H2) focused on technophilia (experience of information technology and medical devices). The results provide partial validation of this hypothesis, as no correlation was observed between IT experience and usability in terms of effectiveness and efficiency. On the other hand, the technophile participants had a significantly better SUS score (satisfaction) than participants with a low level of technophilia. While these results are consistent with those of Harte et al [27], they contradict previous works [11,23]. We explain these results by a relatively homogeneous representation of IT experience as a function of the age of participants in our sample. We believe that these items [51] highlight the subjective representation of technology use (in relation to age) rather than actual performance in the use of hardware. It is possible that older people may feel that they can properly manipulate a tablet without using other features available in the tool. They would then consider themselves to be quite technophilic, as they would be effective in the day-to-day use of the technology. However, their real capacity to adapt to the technologies is unknown. For example, if an update were to be performed on one of the applications commonly used, it is possible that this would destabilize the manipulation carried out by these individuals.

We also observed a correlation between experience with medical devices and usability. However, previous experience in the use of a blood pressure monitor had no impact on usability. Conversely, previous experience in the use of a pulse oximeter had a significant effect on effectiveness. Participants who had previously manipulated a pulse oximeter made significantly fewer errors than those who had never manipulated a pulse oximeter. In contrast, previous experience using a pulse oximeter had no effect on efficiency and satisfaction. All subjects who reported previous use of a pulse oximeter also reported previous manipulation of a blood pressure monitor. This result suggests that prior use of a pulse oximeter in combination with a blood pressure monitor would facilitate manipulation of the Smart Angel device in terms of effectiveness. We believe that participants who are accustomed to using this type of complex device are accustomed to being involved in health issues, which may be evidence of strong patient involvement in their own health [58].

Our third hypothesis (H3) was concerned with the lack of correlation between education level and usability. The results supported our hypothesis, as no significant correlation was found between participants' level of education and usability in terms of effectiveness, efficiency, and satisfaction. These results are also consistent with previous works [11,19,20].

Finally, the fourth hypothesis (H4) postulated that health literacy influences usability (effectiveness, efficiency, and satisfaction). The HLS-EU-Q16 scores showed no effect on usability (Figure 4). In contrast, the NVS scores showed a significant effect on the number of manipulation errors (effectiveness), manipulation time (efficiency), and SUS score (satisfaction). This is consistent with the results of previous studies [18,28,29]. Significant and medium-sized correlations between the NVS score and each of the usability dimensions were observed (Figure 4). This suggests that the higher the literacy level of the participants, the fewer manipulation errors they make (ie, the more effective they are), the faster they are (ie, the more efficient they are), and the higher the SUS score will be (ie, the more satisfied they are). However, after clustering the participants as recommended [40], there is a significant correlation between NVS literacy level and the number of errors (effectiveness) but no correlation with the manipulation time (efficiency) and the SUS score (satisfaction). Participants with a sufficient literacy level made significantly fewer errors than those with inadequate or problematic literacy.

It is important to note that the HLS-EU and NVS results are contradictory and demonstrate the complexity of health literacy assessment. In addition, our results suggest that the HLS-EU questioning the participants’ subjective abilities to access health information and make decisions introduces a significant bias in the measurement of health literacy. Some participants may claim to have no difficulty using health information, but there is no verification that this is, in fact, the case. Conversely, the NVS instrument appears to be better suited to gathering information on subjects' cognitive abilities, as it is a test that collects information on participants' thought processes when reading a food label, thus providing a more objective assessment of health literacy.

### Conclusions and Research Prospects

This study provides theoretical insight into the effects of user characteristics (eg, age, experience, education, and health literacy) through the use of personas with respect to usability (effectiveness, efficiency, and satisfaction, according to ISO 9241-11 [3]) in the case of the Smart Angel connected medical device. This study provides a methodological contribution insofar as it revealed the differences in data collection between the NVS and the HLS-EU-Q16, thus demonstrating the importance of continuing research in the field of health literacy measurement tools. In addition, these results allow us to better understand the importance of the impact of technophilia among older people with a sufficient level of health literacy for usability.

The results of this study suggest 4 research prospects. First, the relevance of the personas method in the prototype evaluation phase has never been proven. This method is classically used in the design phase by designers (ergonomists, designers, engineers, and even future users) but more rarely used in an evaluation framework. To validate this method in this new context of use in the evaluation phase, it would be necessary to reproduce this study by adding a control group (ie, a group for whom the personas are not presented). Secondly, the training carried out by the researcher could be adapted according to the literacy levels of the participants. Indeed, the main difficulty in the use of a medical device is understanding the procedures, and this cannot be achieved if there is insufficient upstream training [59]. Training should certainly be adapted to the ages and literacy levels of the participants. Demonstration by the researcher may be sufficient for groups with adequate levels of health literacy. Conversely, for groups with inadequate or problematic levels of health literacy, further instruction should be considered. Third, the choice of questionnaire is a crucial step in measuring health literacy. Indeed, we observed a significant disparity in results between the HLS-EU-Q16 and the NVS. As already discussed, these 2 questionnaires do not appear to assess the same dimensions of health literacy. Further work is needed to understand what exactly is being assessed by each of the health literacy questionnaires. We believe that it is better to evaluate this skill with objective assessments. In the same way, it would have been interesting to perform objective measurements of technophilia.

Finally, beyond health literacy, it would now be appropriate to measure the level of eHealth literacy [20]. Unfortunately, there is no valid questionnaire in French on this subject. Thus, more systematic translations and adaptations of these tools should be considered in future studies.

Currently, as a result of this study, the Smart Angel device is in clinical trials where usability tests continue to be carried out in in situ conditions.

## Appendix

Multimedia Appendix 1 Example of a persona.

## Abbreviations

HLS-EU: European Health Literacy Survey
HLS-EU-Q16: 16-item European Health Literacy Survey
ICC: intraclass correlation
IT: information technology
NVS: Newest Vital Sign
REALM: Rapid Estimate of Adult Literacy in Medicine
SUS: System Usability Survey
S-TOFHLA: Short Test of Functional Health Literacy in Adults
TOFHLA: Test of Functional Health Literacy in Adults
