# Supplementary material for: Effects of User Characteristics on the Usability of a Home-Connected Medical Device (Smart Angel) for Ambulatory Monitoring: Usability Study
Source: JMIR Hum Factors. 2021 Mar 17;8(1):e24846. doi: 10.2196/24846 (PMC8080268; doi:10.2196/24846)
Supplement: Multimedia Appendix 1 [file humanfactors_v8i1e24846_app1.pdf]

Fatima

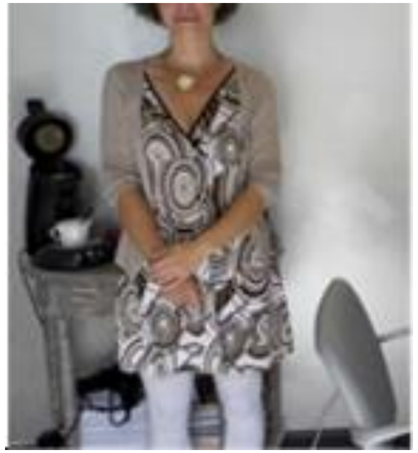

- **Profile**
  - 55 years old, lives alone with her dog. She is a hairdresser and has her own hairdressing salon.
  - Her daughter often comes to see her, she will pick up her mother at the hospital after the surgery.
  - She works a lot; she is a volunteer at the "restos du cœur".
  - A key sentence: "Never at home, always be active".

- **Pleasure/ emotions, behavior**
  - *Sunday is sacred; it's for my granddaughter.*
  - She likes discussions, people, gossip, hikes...
  - She doesn't like emptiness, silence, and demanding customers.
    - She is prudent and organized

Carpal tunnel surgery

- **Technologies**
  - She has a computer in the living room, but it's not handy.
    - She prefers to call rather than text
    - She bought herself a tablet for the photos.
  - She has a website for the hair salon.
  - She follows bloggers and influencers in fashion and hairdressing.

- **Health**
  - She has rheumatism but she takes care of herself.
  - She pays a lot of attention to her well-being
    - Cream, make-up, sauna, beauty care...
  - She pays attention to her diet
    - But she likes sweets
  - She always needs to understand right away
    - (e.g. medical analyses)
  - She is not interested in connected health (e-health).
  - She has low health literacy
    - *If they tell me to take medication, I take it*
  - **Motivations/ Smart Angel**
    - It's comforting for the follow-up and explanations.
      - But could I ask questions?
    - She trusts the medical profession
